# Mcl-1 expression and JNK activation induces a threshold for apoptosis in Bcl-xL-overexpressing hematopoietic cells

**DOI:** 10.18632/oncotarget.14223

**Published:** 2016-12-26

**Authors:** Yu Zhang, Xin Li, Shisheng Tan, Xinyu Liu, Xinyu Zhao, Zhu Yuan, Chunlai Nie

**Affiliations:** ^1^ Departmant of Oncology, Guizhou People's Hospital, Guizhou, China; ^2^ State Key Laboratory of Biotherapy and Cancer Center, West China Hospital, Sichuan University and Collaborative Innovation Center for Biotherapy, Chengdu, P. R. China

**Keywords:** 2-DG, ABT-199, Apoptosis, Bcl-xL, Mcl-1

## Abstract

The regulation of Mcl-1 expression is necessary for the induction of cancer cell apoptosis by ABTs such as ABT-737, ABT-263 and ABT-199. However, the reduction in Mcl-1 expression is not sufficient for initiating cell death in hematopoietic cancer cells with high Bcl-xL expression. Here, we demonstrate that 2-deoxyglucose (2-DG) enhanced the effect of ABT-199 to induce cell apoptosis in hematologic malignancies with up-regulated Bcl-xL expression. Our study revealed that 2-DG could decrease glucose-dependent and Akt-independent Mcl-1 expression, which is mediated by the mechanistic target of rapamycin complex 1 (mTORC1) pathway. Moreover, the combination of 2-DG and ABT-199 triggered c-Jun NH2-terminal kinase (JNK) phosphorylation and subsequent Bcl-xL degradation, whereas 2-DG and ABT-199 alone had little effect on JNK activation. Therefore, the combination of 2-DG and ABT-199 initiated cell death through the reduction of Mcl-1 expression and JNK activation. Our study could provide a clinical theoretical basis for the use of ABT-199 in hematologic malignancies with excessive Bcl-xL expression.

## INTRODUCTION

The overexpression of anti-apoptotic Bcl-xL, Mcl-1 or Bcl-2 occurs frequently in cancers, particularly in hematologic malignancies such as acute myeloid leukemia (AML) and multiple myeloma (MM), resulting in defective apoptosis leading to enhanced cell survival and drug resistance [1, 2]. The anti-apoptotic Bcl-2 family proteins protect against apoptosis by neutralizing the function of pro-apoptotic Bcl-2 family members such as Bax and Puma and by preventing both the release of cytochrome c from mitochondria and subsequent apoptotic events, such as caspse-3 activation [3]. Bcl-xL up-regulation is responsible for ABT-737 resistance in chronic lymphocytic leukemia [4]. Moreover, the overexpression of the Bcl-xL protein contributes to tumor cell survival in lymphoid leukemia that can be treated by ABT-199 [5]. Therefore, abrogating or overcoming the anti-apoptotic function of Bcl-xL protein may increase chemosensitivity and reverse chemoresistance in hematopoietic tumor cells.

ABT-263 or ABT-737, BH3 mimetics with a high affinity for both Bcl-2 and Bcl-xL, are used to overcome the chemoresistance mediated by Bcl-2 or Bcl-xL up-regulation *in vitro* and *in vivo* [6, 7]. The doses of these two agents that can be used clinically are limited by the accompanying thrombocytopenia, which is caused by the inhibition of Bcl-xL in platelets [8, 9]. To address this problem, ABT-199, a more selective ABT-263 derivative that specifically binds Bcl-2, was designed [9]. ABT-199 could induce cell death in Bcl-2-overexpressing hematopoietic cancer cells [9–12]. However, ABT-199 is not efficient for cancer cells with excessive Bcl-xL expression [5, 10–13]. Thus, it is necessary to determine a way to overcome the Bcl-xL chemoresistance in cancer cells.

In this study, we first revealed that 2-deoxyglucose (2-DG), a glycolytic inhibitor, combined with ABT-199 triggered apoptosis in AML, MM and lymphoid cells with high Bcl-xL expression. We found that ABT-199 or 2-DG alone could not induce apoptosis in cells with high Bcl-xL expression. We then determined the molecular mechanism of apoptosis induced by ABT-199 and 2-DG. Our study demonstrated that 2-DG treatment initiated glucose-dependent and Akt-independent Mcl-1 degradation, which is regulated by the mechanistic target of rapamycin complex 1 (mTORC1) pathway. Mcl-1 degradation contributed to the apoptosis induced by ABT-199 and 2-DG. Moreover, 2-DG and ABT-199 treatment led to JNK activation, which induced Bcl-xL phosphorylation and degradation in cells. ABT-199 or 2-DG alone did not trigger JNK activation. Bcl-xL degradation could promote the cell death induced by ABT-199 and 2-DG. Thus, the combination of 2-DG and ABT-199 overcame the Bcl-xL-mediated apoptosis chemoresistance through two signaling pathways.

## RESULTS

### Combination treatment of 2-DG and ABT-199 induces apoptosis in hematopoietic cancer cells with high Bcl-xL expression

**Figure 1:**
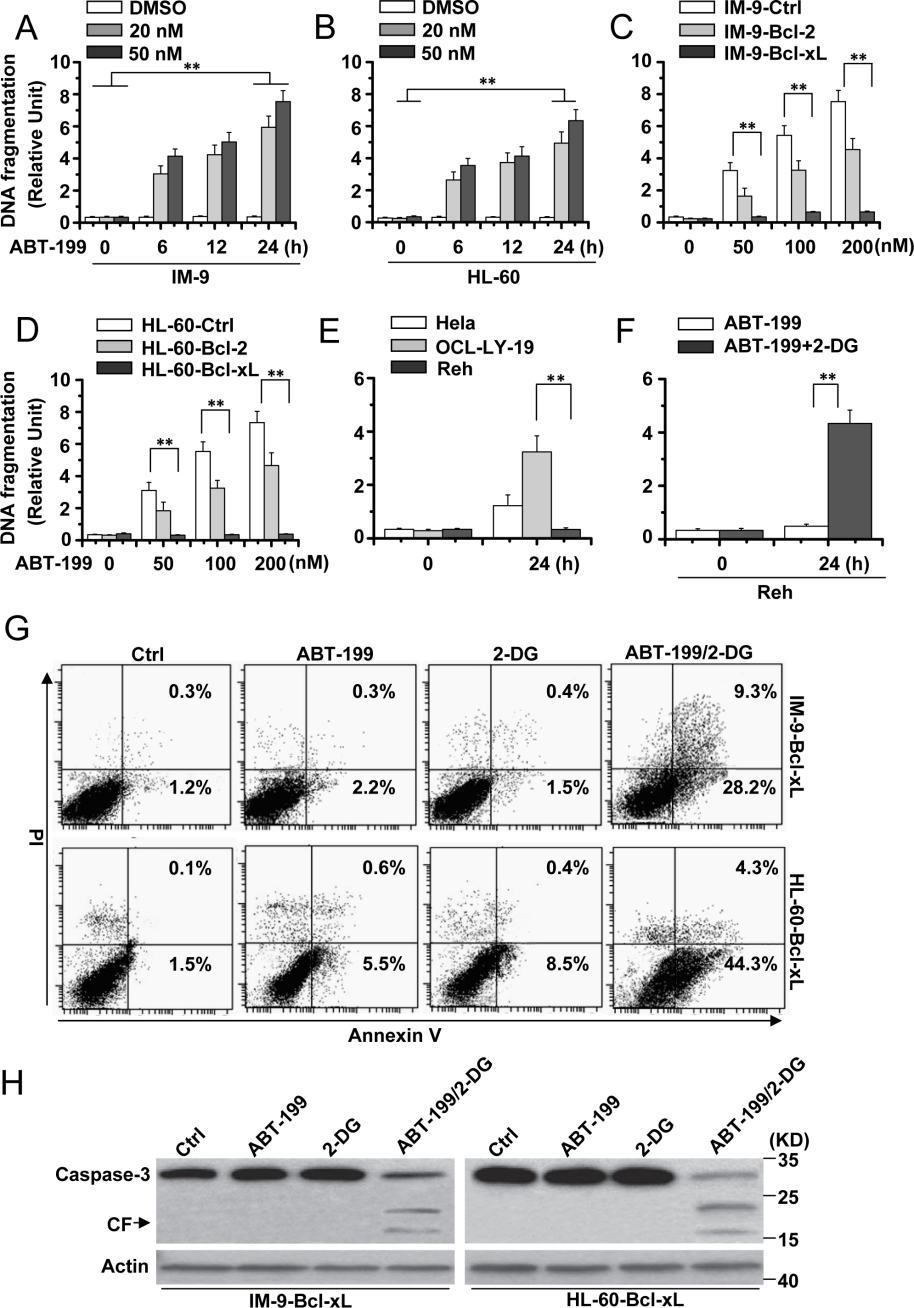
2-DG combined with ABT-199 induces cell apoptosis in hematopoietic cancer cells with excessive Bcl-xL expression (**A**) and (**B**) Analysis of cell apoptosis treated with ABT-199. IM-9 and HL-60 cells were treated with indicated concentrations of ABT-199 for different periods of time and then collected to examine apoptosis. Cell apoptosis was quantitatively detected by a cell death ELISA kit as described in Materials and methods. Graphs showing results of quantitative analyses (*n* = 3, mean ± S.D. **P < 0.01); (**C**) IM-9 cells were stably transfected with Ctrl, Bcl-2 or Bcl-xL vector and then treated with different concentrations of ABT-199 for 24 h. Treated cells were lysed for apoptosis detection as described in A. Graphs showing results of quantitative analyses (*n* = 3, mean ± S.D. **P < 0.01); IM-9-Bcl-2 or IM-9-Bcl-xL refer to overexpressing Bcl-2 or Bcl-xL IM-9 cells. (**D**) HL-60 cells were stably transfected with Ctrl, Bcl-2 or Bcl-xL vector and then treated as described in C. Graphs showing results of quantitative analyses (*n* = 3, mean ± S.D. **P < 0.01); HL-60-Bcl-2 or HL-60-Bcl-xL refer to overexpressing Bcl-2 or Bcl-xL HL-60 cells. (**E**) Indicated cells were treated with ABT-199 (50 nM) for 24 h, and then treated cells were collected for apoptosis detection. Graphs showing results of quantitative analyses (*n* = 3, mean ± S.D. **P < 0.01). (**F**) Reh cells were treated with ABT-199 (50 nM) or ABT-199 (50 nM) with 2-DG (5 mM) for 24 h. Treated cells were collected for apoptosis detection. Graphs showing results of quantitative analyses (*n* = 3, mean ± S.D. **P < 0.01). (**G**) Indicated cells were treated with 2-DG, ABT-199 (50 nM) or the combination of the two (2-DG, 5 mM; ABT-199, 50 nM) for 24 h, and then collected for Annexin V and PI double staining with flow cytometry. (**H**) Cells were treated as describe in G for 24 h, and then lysed for western blot detection. β-Actin was used as a protein loading control. Representative results of three experiments with consistent results are shown.

We first determined the apoptotic effects of ABT-199 in MM (IM-9) and AML cell lines (HL-60). We treated the cells with ABT-199 for the indicated time periods, and apoptosis was assessed by a DNA fragmentation ELISA assay. As depicted in Figure 1A and 1B, ABT-199 efficiently induced cell death in IM-9 and HL-60 cells. We then detected the effect of ABT-199 on cells with Bcl-2 or Bcl-xL overexpression. Immunoblotting experiments confirmed the expression of Bcl-2 or Bcl-xL in stably transfected cancer cells (Supplementary Figure 1A). ABT-199 still induced apoptosis in cells with high levels of exogenous Bcl-2 protein, but not in cells with high expression of exogenous Bcl-xL (Figure 1C and 1D), as described before [10].

To better detect the apoptotic effect of 2-DG and ABT-199, we used OCL-LY-19 (low endogenous Bcl-xL expression) and Reh (high endogenous Bcl-xL expression) lymphoid cells [5] (Supplementary Figure 1B). ABT-199 could induce apoptosis in OCL-LY-19 but not Reh cells (Figure 1E), as described before [5]. 2-DG obviously enhanced the apoptosis induced by ABT-199 in Reh cells (Figure 1F).

Flow cytometry analysis with Annexin V/PI staining revealed that the combination of 2-DG and ABT-199 efficiently induced apoptosis in cells with up-regulated Bcl-xL expression, although 2-DG and ABT-199 alone had little effect on cell death (Figure 1G). Further immunoblotting experiments also showed that caspase-3 was activated upon treatment with 2-DG and ABT-199 (Figure 1H). These results indicate that 2-DG combined with ABT-199 could cause apoptosis in hematopoietic cancer cells with high-level expression of Bcl-xL.

### 2-DG-mediated Mcl-1 degradation partially contributed to apoptosis by 2-DG and ABT-199 in cells with high Bcl-xL expression

**Figure 2:**
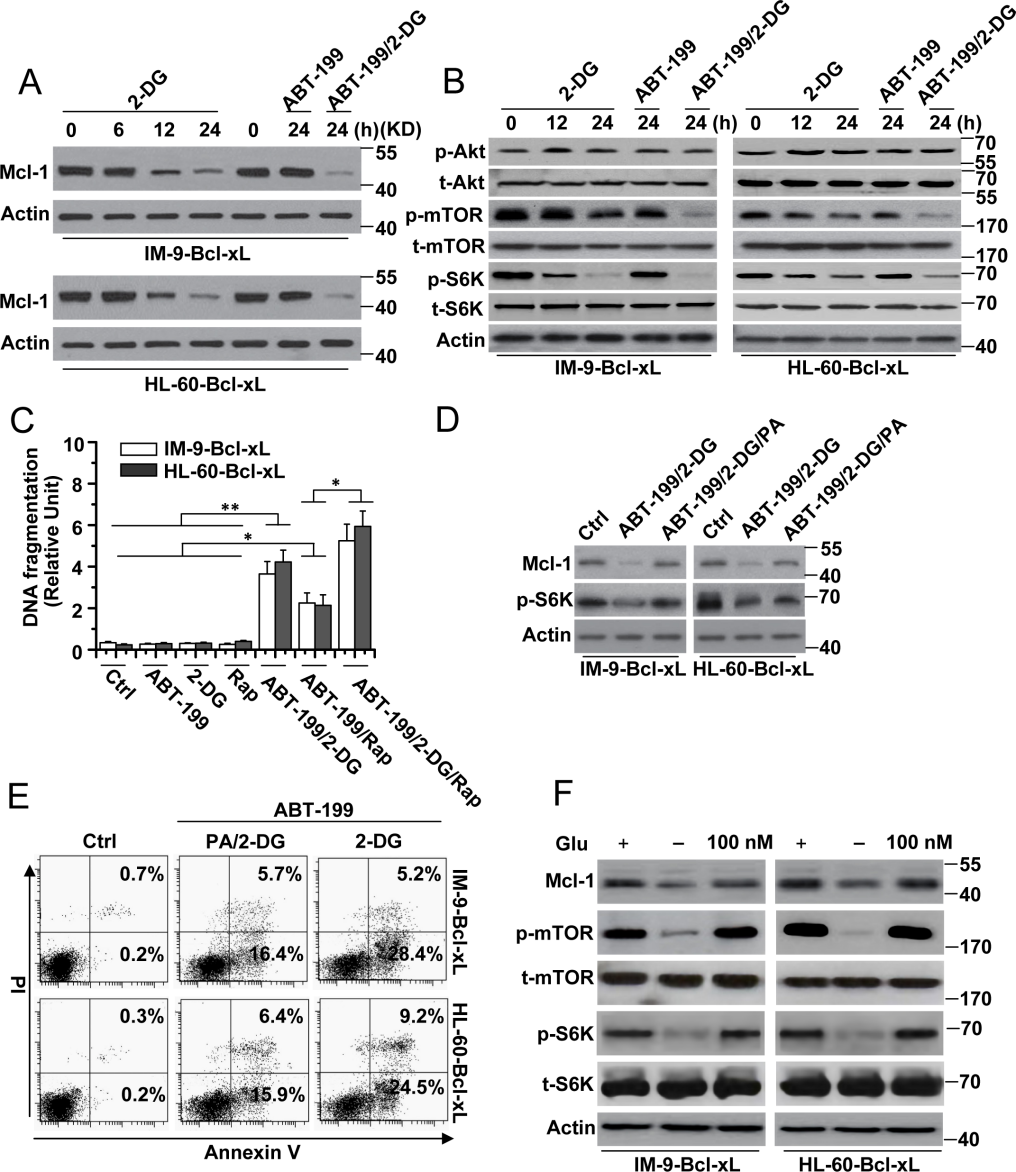
The reduction of Mcl-1 expression is indispensable for cell apoptosis by 2-DG with ABT-199 treatment (**A**) Cells were treated with 2-DG (5 mM), ABT-199 (50 nM) or the combination of the two (2-DG, 5 mM; ABT-199, 50 nM) at the indicated time points, and then lysed for western blot detection. β-Actin was used as a protein loading control. (**B**) Cells were treated as described in A and analyzed for immunoblot. (**C**) Cells were treated with 2-DG (5 mM), ABT-199 (50 nM), rapamycin (25 nM) or the indicated combination treatment for 24 h. Treated cells were collected for apoptosis detection. Graphs showing results of quantitative analyses (*n* = 3, mean ± S.D. *P < 0.05; **P < 0.01). (**D**) Cells were subjected to the indicated combination treatment (2-DG, 5 mM; ABT-199, 50 nM; PA, 100 μM) for 24 h, and then lysed for immunoblot detection. (**E**) Cells were treated as described in D and then collected for Annexin V and PI double staining with flow cytometry. (**F**) Cells were culture in glucose-free RPMI-1640 media as indicated for 24 h, and then added 100 nM glucose for 24. Treated cells were analyzed for immunoblot. All data are representative of three independent experiments.

Because Mcl-1 expression contributes to 2-DG-induced cell death [14, 15], we speculated that the regulation of Mcl-1 expression could mediate the cell death induced by 2-DG and ABT-199. Our experiments revealed that treatment with 2-DG alone or 2-DG with ABT-199 for different periods of time decreased Mcl-1 expression (Figure 2A). ABT-199 alone could not reduce Mcl-1 expression. The same results were also observed in Reh cells (Supplementary Figure 1C). Further experiments revealed that mTOR and S6K phosphorylation was decreased after treatment with 2-DG alone or 2-DG and ABT-199 but not ABT-199 alone (Figure 2B), indicating that the mTORC1-S6K pathway was restrained. It should be noted that no obvious change was detected in Akt phosphorylation after treatment, suggesting that Akt is not affected by treatment with 2-DG and ABT-199.

To validate whether the inhibition of mTOR pathway activity is responsible for the reduced Mcl-1 expression and cell death in our study, we first used rapamycin, an mTOR kinase inhibitor [16], to treat cells. We found that rapamycin alone and in combination with ABT-199 could decrease Mcl-1 expression or mTOR and p62 phosphorylation but that it alone could not cause cell apoptosis (Figure 2C and Supplementary Figure 1D). Meanwhile, rapamycin in combination with ABT-199 obviously induced cell death and enhanced the cell apoptosis induced by 2-DG and ABT-199 (Figure 2C).

We also used phosphatidic acid (PA), which is an endogenous activator of the mTOR kinase [16, 17]. PA treatment efficiently restored Mcl-1 expression in cells treated with 2-DG and ABT-199 (Figure 2D). However, PA treatment only partially reduced the cell apoptosis induced by 2-DG and ABT-199 (Figure 2E). Meanwhile, we found that rapamycin could block the effect of PA on Mcl-1 expression (Supplementary Figure 1E) and apoptosis (data not shown). These results have demonstrated that the mTOR pathway, which is regulated by 2-DG, is involved in the cell death induced by 2-DG and ABT-199.

To rule out the pro-autophagic effects of the mTOR pathway, we examined the expression of p62, a protein that can be degraded by rapamycin during autophagy [18]. As described in Supplementary Figure 2A, the expression of p62 was little changed with the treatment of either ABT-199 or 2-DG alone or the combination of the two in cells with high Bcl-xL expression. Meanwhile, rapamycin alone or in combination with ABT-199 also had little effect on p62 expression in cells with high Bcl-xL expression (Supplementary Figure 2B). IM-9 cells were used as a control. We then reduced the p62 expression by transfecting the cells with p62-specific siRNA (Supplementary Figure 2C). We found that p62 siRNA could not affect the cell apoptosis induced by ABT-199 combined with either 2-DG or rapamycin (Supplementary Figure 2D). These results indicated that the mTOR pathway could not cause autophagy in cells with Bcl-xL overexpression.

We then determined whether glucose was involved in Mcl-1 expression, as a previous study revealed that the 2-DG-mediated mTOR pathway is dependent on glucose metabolism [14]. Glucose deprivation led to decreased Mcl-1 expression and mTOR and S6K phosphorylation, whereas 100 nM glucose restored Mcl-1 expression and mTOR and S6K phosphorylation in cells (Figure 2F). These results revealed that 2-DG-mediated glucose deprivation indeed regulated the mTOR pathway and Mcl-1 expression. However, glucose deprivation alone could not induce cell apoptosis. Moreover, glucose deprivation with ABT-199 induced a small amount of apoptosis (data not shown). These results indicate that 2-DG-induced glycolysis–dependent mTOR signaling is only one pathway that mediates apoptosis and that there are other pathways involved in apoptosis in cells with high-level expression of Bcl-xL.

### JNK-mediated Bcl-xL phosphorylation and degradation is important for apoptosis in cells with high-level expression of Bcl-xL

**Figure 3:**
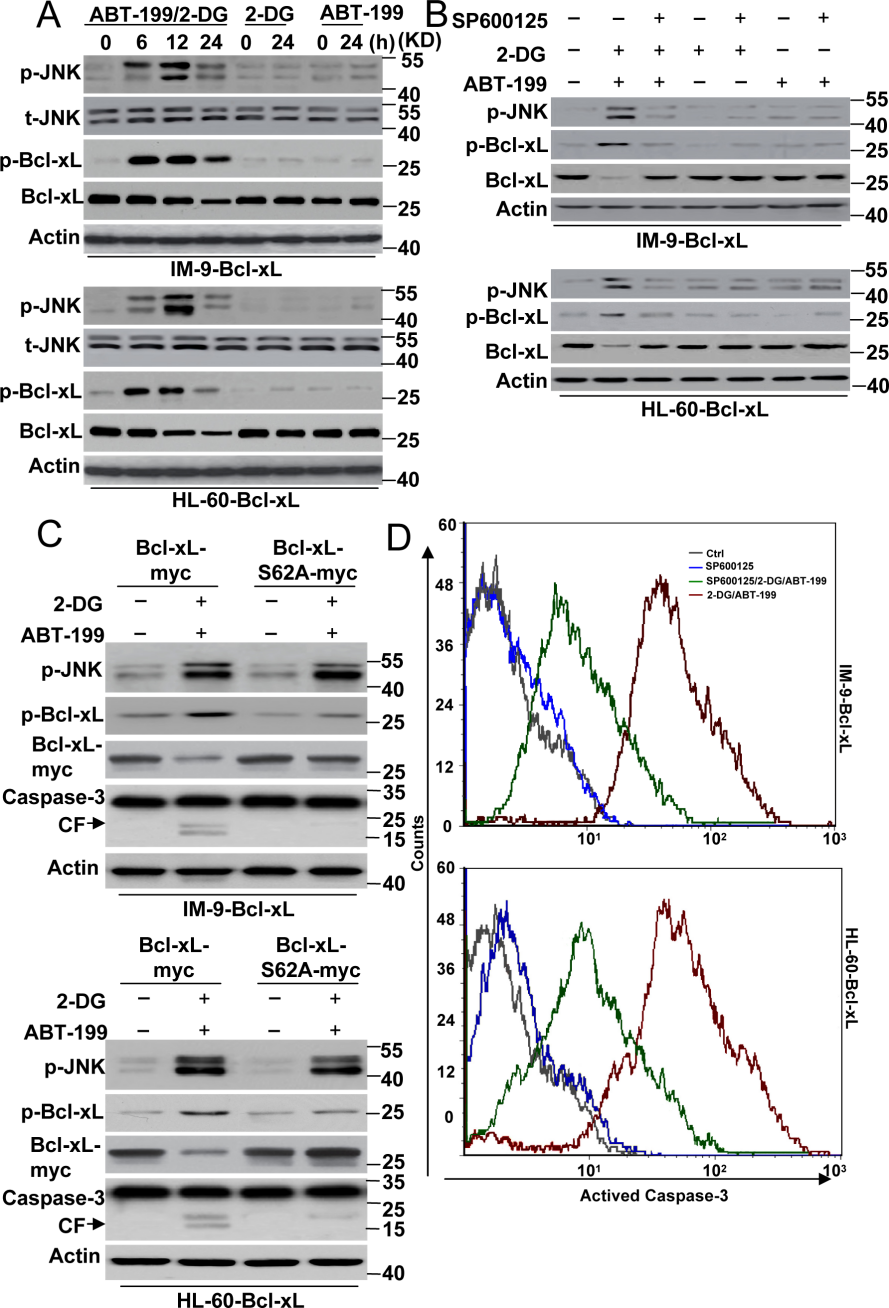
JNK mediated Bcl-xL degradation contributes to cell death by 2-DG with ABT-199 treatment (**A**) Cells were treated with 2-DG (5 mM), ABT-199 (50 nM) or the combination of the two (2-DG, 5 mM; ABT-199, 50 nM) at the indicated time points, and then lysed for western blot detection. β-Actin was used as a protein loading control. (**B**) Cells were treated with 2-DG (5 mM), ABT-199 (50 nM), SP600125 (20 μM) or the indicated combination treatment for 24 h, and then lysed for western blot detection. (**C**) Cells were transfected with Bcl-xL-Myc or Bcl-xL-S62A-Myc for 48 h, and then cells were treated with 2-DG and ABT-199 for 24 h. Treated cells were lysed for immunoblot detection. (**D**) Cells were treated as described in B, and then collected for caspase-3 activity detection with a special antibody for cleaved caspase-3 (#9664, Cell Signaling) by flow cytometry. Representative results of three experiments with consistent results are shown.

Previous studies have demonstrated that JNK is a stress-activated protein kinase that is activated in response to ER stress [19, 20], which could be regulated by 2-DG [21]. Moreover, ABT-199 has been reported to induce the rapid phosphorylation of JNK kinase in follicular lymphoma cells. The inhibition of JNK activation further augmented the apoptosis induced by ABT-199 [22, 23]. These results indicate that JNK is a target of 2-DG or ABT-199. We then determined whether JNK contributed to cell apoptosis in our study. We assayed the status of JNK activation by western blot analysis. JNK phosphorylation was visible starting at 6 h of treatment with 2-DG and ABT-199 and peaked at 12 h of treatment (Figure 3A and Supplementary Figure 3A). 2-DG or ABT-199 alone had little effect on JNK activation in cells with up-regulated Bcl-xL expression.

A western blot assay also revealed that Bcl-xL phosphorylation, concomitant with the activation of JNK, was suggestive of a causative role of this kinase (Figure 3A). It should be noted that Bcl-xL expression was decreased (Figure 3A and Supplementary Figure 1C), suggesting that phosphorylated Bcl-xL is a target for protease degradation, as indicated by previous studies [24, 25]. Indeed, our experiments showed that MG 132, a widely distributed protease inhibitor, efficiently restored Bcl-xL expression (Supplementary Figure 3B). Further experiments found that a JNK inhibitor, SP600125, or JNK1-specific siRNA almost completely prevented the phosphorylation and degradation of Bcl-xL, thus suggesting a role for JNK in Bcl-xL phosphorylation and degradation (Figure 3B, Supplementary Figure 3C and 3D).

Ser 62 is major phosphorylation site of Bcl-xL [26]. The mutation of Ser 62 prevents Bcl-xL phosphorylation. We constructed a Bcl-xL mutant in which Ser 62 was changed to Ala (S62A) (Supplementary Figure 4A) and transfected this mutant into cells. As depicted in Figure 3C, the Bcl-xL S62A mutant decreased Bcl-xL phosphorylation and degradation compared with the wild-type Bcl-xL (WT). Meanwhile, JNK phosphorylation was not affected by the Bcl-xL mutant after treatment, further indicating that JNK is the upstream kinase regulating the function of Bcl-xL in cells.

We then detected whether the JNK-Bcl-xL pathway contributed to cell apoptosis. Our data revealed that 2-DG and ABT-199 induced obvious caspase-3 cleavage in cells with Bcl-xL WT expression, whereas caspase-3 cleavage was not visible in cells with Bcl-xL S62A mutant expression (Figure 3C). Moreover, SP600125 also decreased caspase-3 activity after 2-DG and ABT-199 treatment (Figure 3D). These results indicated that JNK-mediated Bcl-xL phosphorylation and degradation is required for cell apoptosis. However, it is worth noting that neither SP600125 treatment nor the Bcl-xL mutant could completely inhibit apoptosis, suggesting that the JNK-Bcl-xL pathway is not the only signal pathway regulating cell apoptosis in Bcl-xL-overexpressing cells.

### Mcl-1 expression and JNK activation cooperatively induce cell apoptosis in cells with high Bcl-xL expression

**Figure 4:**
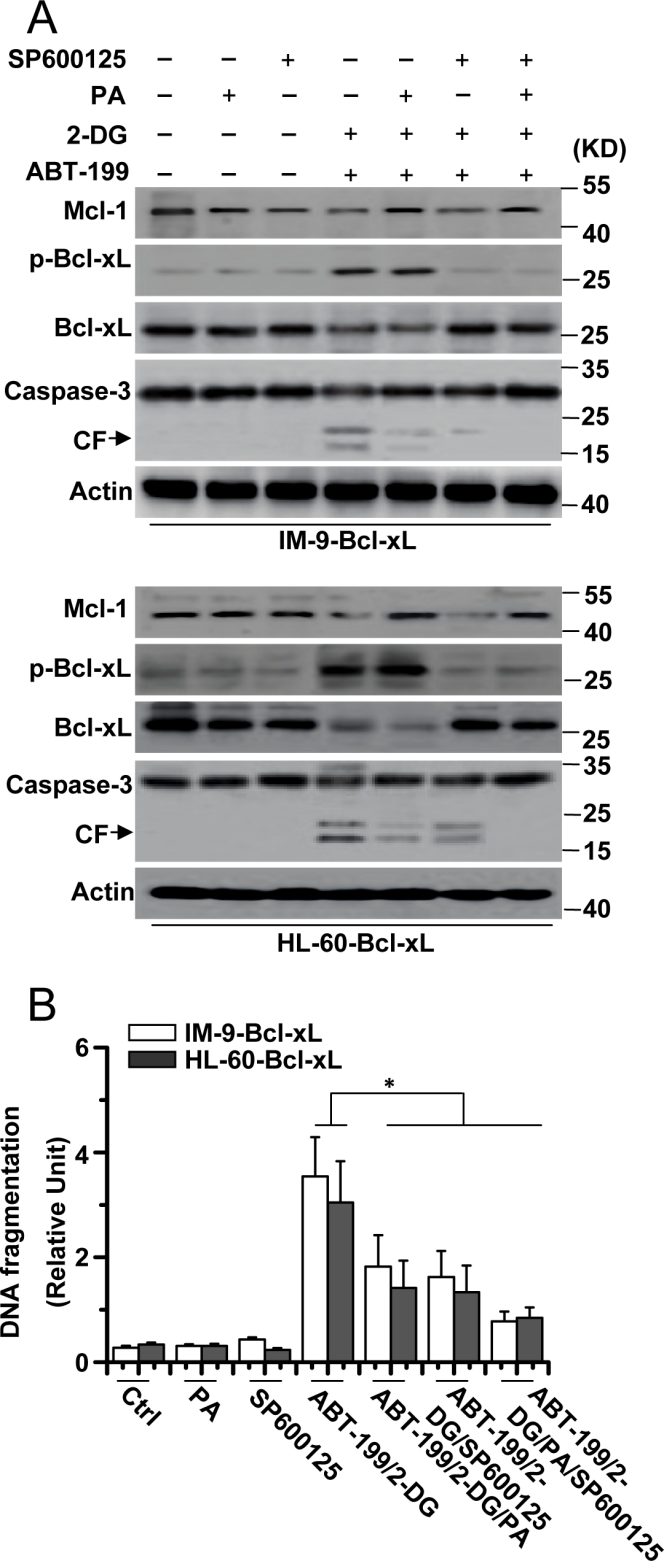
Mcl-1 expression and JNK activation is required for cell apoptosis (**A**) Cells were treated with 2-DG (5 mM), ABT-199 (50 nM), SP600125 (20 μM), PA (100 mM) or the indicated combination treatment for 24 h, and then lysed for western blot detection. β-Actin was used as a protein loading control. (**B**) Cells were treated as described in A, and then collected for apoptosis detection. Graphs showing results of quantitative analyses (*n* = 3, mean ± S.D. *P < 0.05). All data are representative of three independent experiments.

We then tested whether Mcl-1 expression and JNK activation cooperatively mediated cell death. PA and SP600125 efficiently inhibited caspase-3 cleavage after 2-DG and ABT-199 treatment (Figure 4A). Meanwhile, PA and SP600125 co-treatment greatly reduced the apoptosis of the cells (Figure 4B). PA affected Mcl-1 expression but not Bcl-xL phosphorylation and degradation after 2-DG and ABT-199 treatment, whereas the effect of SP600125 was just the opposite of PA treatment. Moreover, glucose deprivation and mTOR inhibition, which affect Mcl-1 expression, had no effect on Bcl-xL expression (Supplementary Figure 4B). These results demonstrated that Mcl-1 expression and JNK activation triggered apoptosis in cells with up-regulated Bcl-xL expression.

## DISCUSSION

Glucose metabolism is important for tumorigenesis and cancer chemoresistance [27–29]. 2-DG, a glycolytic inhibitor, has been reported to enhance ABT-737/263-induced cell apoptosis *in vitro* and *in vivo* [14, 15, 30]. Moreover, the synergistic effect of 2-DG with ABT-737 or ABT-263 has been reported to be more effective than single drug treatment at promoting the survival of animals transplanted with lymphoma or prostate cancer [31]. In clinical trials, the combination of 2-DG and ABT-737 has the potential to be used in treating ABT-737 resistance [30]. Previous studies have shown that 2-DG could also enhance the cell death effect of ABT-199 on MM cells [32, 33]. However, the detailed mechanism is still unknown.

In this study, we first provide evidence that the combination of 2-DG and ABT-199 induces apoptosis in cells with up-regulated Bcl-xL expression. Since ABT-199 is a potential agent for hematologic malignancies, it is necessary to expand the range of its treatment. However, ABT-199 has little effect on Bcl-xL-overexpressing cancer cells [10, 11]. We attempted to use 2-DG and ABT-199 for the treatment of cancer cells with Bcl-xL overexpression. Surprisingly, we found that the combination of 2-DG and ABT-199 could induce cell apoptosis. Previous studies have proposed two mechanisms explaining the effect of 2-DG on ABT-263/737-induced apoptosis. In the first mechanism, 2-DG decreases Mcl-1 levels indirectly by inhibiting glycolysis, leading to the activation of AMP-activated protein kinases, such as mTORC1, and the inhibition of Mcl-1 synthesis [14, 34]. In the second mechanism, 2-DG weakens the interaction between Bak and Mcl-1, which increases the ability of ABT-263/737 to release Bak from the Mcl-1/Bcl-xL/Bak heterotrimer, thus inducing apoptosis [30, 35]. In fact, the reduction of Mcl-1 expression definitely weakens the link between pro- and anti-apoptotic Bcl-2 family proteins, such as Noxa/Mcl-1 [36] and Bim/Mcl-1 [14], resulting in the release of pro-apoptotic Bcl-2 proteins. Moreover, previous studies also revealed that either Noxa or Bim was activated, accompanied by a reduction in Mcl-1 expression, after treatment with 2-DG alone or the combination of 2-DG and other agents, such as ABT-737 [14, 36]. Yamaguchi and his colleagues revealed that the combination of 2-DG and ABT-737/263 could not decrease Mcl-1 expression but could initiate Bid activation, priming cells for ABT737/263-induced apoptosis [35]. Together, these results indicate that Mcl-1 is a central molecule for the cell apoptosis induced by the combination of 2-DG and ABT-737/263.

Our data also revealed that 2-DG reduced glucose-dependent Mcl-1 expression, consistent with a previous study [14]. Moreover, the reduction in Mcl-1 expression contributes to cell apoptosis. In contrast to previous results [14, 36], we did not observe obvious changes in the expression of Noxa or Bim in our study (Supplementary Figures 1C and 4C). Our findings also revealed that Bid was activated, consistent with the data from Yamaguchi and his colleagues [35]. However, Bid depletion had little effect on the apoptosis induced by 2-DG and ABT-199 (data not shown). Moreover, our experiments revealed that the depletion of Mcl-1 with siRNA (Supplementary Figure 5A) only partially enhanced the apoptotic effect of ABT-199 (Supplementary Figure 5B and 5C), whereas 2-DG obviously increased cell apoptosis. These results indicate that Mcl-1 signaling is not the sole key pathway for the induction of apoptosis by 2-DG and ABT-199.

Yamaguchi and his colleagues revealed that the combination of 2-DG and ABT737/263 could induce apoptosis in cells with ABT737/263 resistance. They used HeLa cells as an initial ABT-737/263-resistant cell model. We used Bcl-xL-overexpressing cells. In fact, HeLa cells express a relatively small amount of Bcl-xL [37] (Supplementary Figure 1B). Our and other group's report [5] has revealed that high Bcl-xL expression in hematopoietic cells inhibited the apoptotic effect of ABT-199. A previous study also confirmed that Bcl-xL expression in acquired ABT-199-resistant lymphoid cell lines was higher than that in ABT-199-sensitive cells [5]. However, the Bcl-xL expression in acquired ABT-737-resistant lymphoid cell lines was very low [37]. These results reveal that the levels of Bcl-xL expression are important for ABT-199 resistance in hematopoietic cells but not for ABT-737/263 resistance. Therefore, we need to not only regulate Mcl-1 but also Bcl-xL expression in hematopoietic cells to overcome ABT-199 resistance.

Our experiments revealed that the combination of 2-DG and ABT-199 also initiated another pathway to induce apoptosis. The second pathway is JNK-mediated Bcl-xL phosphorylation and degradation, which leads to the further release of pro-apoptotic Bcl-2 proteins. As described before [10, 36], 2-DG or ABT-199 alone could not affect Bcl-xL expression in cancer cells. However, our data showed that the combination of 2-DG and ABT-199 could trigger JNK-dependent Bcl-xL degradation and cell death. 2-DG or ABT-199 alone also had little effect on JNK activation, although ABT-199 could induce JNK activation in hematopoietic cancer cells [22, 23]. We speculate that the overexpression of Bcl-xL raises the threshold for JNK activation and cell death. As shown in previous studies, neither 2-DG treatment to inhibit glycolysis nor ABT-737 treatment alone was sufficient to cause cell death in ABT-737-resistant cell lines. A combination of ABT-737 and 2-DG caused significant cell death in resistant cells [14]. Thus, simple agents (ABTs or 2-DG) could reach the threshold of apoptosis in resistant cancer cells. Of course, this requires that we continue making great efforts and study further mechanisms of JNK activation. After all, an elevated phosphorylation level of Bcl-2 has been observed following ABT-199 treatment, which could be regulated by JNK activation [38, 39].

A previous study also revealed that activated Akt contributed to ABT-199 resistance in hematopoietic cells [5]. However, our data demonstrated that Akt was not activated after treatment with either 2-DG or ABT-199 alone or the combination treatment. Rapamycin could induce Akt dephosphorylation (Supplementary Figure 6), which leads to Akt inactivation. However, rapamycin alone could not induce cell death in Bcl-xL-overexpressing cells. Thus, although Akt is important for Mcl-1 expression, Akt is not required for regulating the mTORC1 pathway, as previously reported [14]. 2-DG could decrease intracellular ATP levels, which act directly on the mTOR pathway [40]. Meanwhile, we exclude the effect of autophagy by the mTOR pathway on apoptosis. Rapamycin could decrease p62 expression during autophagy and cause autophagy [18]. However, we observed little change in p62 expression in cells with high Bcl-xL expression after treatment with rapamycin alone or in combination with ABT-199, although rapamycin reduced p62 expression in IM-9 cells. These findings are in agreement with a previous study demonstrating that Bcl-xL expression enhanced the connection between Bcl-xL and Beclin-1, resulting in the inhibition of autophagy [41]. Moreover, our and previous findings [15, 35] revealed that 2-DG enhanced the apoptotic effect of ABTs through Bcl-2 family proteins, which mediate the mitochondrial apoptosis pathway. Thus, an extrinsic apoptotic pathway such as caspase-8 may not be necessary for our observed results, although p62 could contribute to an extrinsic apoptotic pathway through regulating caspase-8 activity [42].

In summary, we first report that 2-DG and ABT-199 could induce cell apoptosis in hematopoietic cancer cells with Bcl-xL overexpression. Moreover, we present evidence to show that in addition to the traditional Mcl-1 pathway, the JNK pathway is also activated after 2-DG and ABT-199 treatment. These two signal pathways overcome Bcl-xL chemoresistance to initiate cell apoptosis. Our findings suggest a novel mechanism that modulates the expression and activity of pro-survival proteins to confer treatment resistance that could be exploited by a rational combination therapeutic regimen effective for treating hematopoietic malignancies.

## MATERIALS AND METHODS

### Materials

2-DG, PA, SP600125, glucose, rapamycin, the proteasome inhibitor MG132, Annexin V and PI were obtained from Sigma (St. Louis, MO, USA). ABT-199 was from Selleck Chemicals (Houston, TX, USA). myc (clone 9E10, M4439) and actin (clone AC-74, A5316) antibodies were also from Sigma. phospho-Akt (Ser 473) (clone 587F11, #4051), Akt (#9272), phosphor-mTOR (Ser2448)(#5536), mTOR (#2972), phosphor-p70S6K (Thr389)) (#9234), p70S6K (#9202), phospho-JNK (Thr 183/Tyr 185) (#9251), JNK (#9252), caspase-3 (clone 8G10, #9665), and Mcl-1 (#4572) antibodies were purchased from Cell Signaling (Beverly, MA). Bcl-2 (sc-7382), Bcl-xL (sc-8392), phosphor-Bcl-xL (Ser 62) (sc-101644), BimEL (sc-27982), p62(sc-55603), Noxa (sc-56169) and Bid (sc-56025) antibodies were from Santa Cruz (Santa Cruz, CA).

### Plasmids and gene silencing with small interfering RNAs

pEF MYC(hs) Bcl-2 puro [43] are a gift from David C.S. Huang (Walter And Eliza Hall Institute For Medical Research, Victoria, Australia) and pCDNA3.1-Bcl-xL [44], which is a gift from Richard J Youle (Biochemistry Section, Surgical Neurology Branch, National Institute of Neurological Disorders and Stroke, National Institutes of Health, Bethesda, MD, USA). These constructs were subcloned into into the pcDNA 3.1/myc-His A vector (Invitrogen, Carlsbad, CA, USA) to product the Bcl-2-myc or Bcl-xL-myc constructs. Bcl-xL (S62A) was generated by site-directed mutagenesis using Pfu-ultra poly-merase (Stratagen, La Jolla, CA, USA) followed by DpnI digestion (Fermentas Inc., Glen Burnie, MD, USA) according to the manufacturer's instructions. Human Mcl-1 small interfering RNA (siRNA; sc-35877), p62 (siRNA; sc-35232) or JNK1 (sc-29380), which consists of a pool of three target-specific 19 to 25 nt siRNA and the nonsilencing control siRNA (sc-37007) was purchased from Santa Cruz Biotechnology, Inc.

### Cell culture and transfection

IM-9 and HL-60 cells were obtained from the American Type Culture Collection (Rockville, MD). Cells were cultured with RPMI-1640 media (Sigma) supplemented with 10% fetal bovine serum (Hyclone, Logan, UT, USA) and 1% penicillin–streptomycin at 37°C under 5% CO_2_ FBS. Glucose starvation was accomplished by washing 3× with PBS and culture in glucose-free RPMI-1640 media (Sigma) with 10% dialyzed FBS (Gemini BioProducts).

For transfection, cells were seeded on 6-well plates and then transfected with the appropriate siRNA or plasmids using the manufacturer's protocols. Typically, cells were seeded on coverslips in the 6-well plates, and then 100 nM siRNA and 4 μl of DMRIE-C reagent (Invitrogen, Carlsbad, CA, USA) were used per coverslip. The cells were incubated for 4 h in the transfection mixture, which was then replaced with fresh culture medium. For getting the IM-9-Bcl-2, IM-9-Bcl-xL, HL-60-Bcl-2 and HL-60-Bcl-xL cells, IM-9 and HL-60 cells were stably transfected with pcDNA 3.1-Bcl-2 or Bcl-xL.

### Apoptosis assays

Three methods were used to assess apoptotic cell death: detection of DNA fragmentation with the Cell Death Detection ELISA kit (Roche Diagnostics), Western blot analysis of caspase-3 cleavage and measurement of apoptotic cells by flow cytometry (Annexin/PI [45] or activated caspase-3). The Cell Death Detection ELISA quantified the apoptotic cells by detecting the histone-associated DNA fragments (mono- and oligo-nucleosomes) generated by the apoptotic cells [3, 46].

### SDS-PAGE and immunoblotting

SDS-PAGE and immunoblotting were performed as described before [47]. Briefly, the cells or the membrane fractions were resuspended in a lysis buffer containing Nonidet P-40 (10 mM Hepes, pH 7.4, 2 mM EGTA, 0.5% Nonidet P-40, 1 mM NaF, 1 mM NaVO4, 1 mM phenylmethylsulfonyl fluoride, 1 mM dithiothreitol, 50 μg/ml trypsin inhibitor, 10 μg/ml aprotinin, and leupeptin) and were placed on ice for 30 min. The lysates were centrifuged at 12,000 × g for 12 min at 4°C, and the protein concentration was measured. Equivalent samples (30 or 50 μg of protein) were subjected to SDS-PAGE on 12% gels. The proteins were then transferred onto nitrocellulose membranes and probed with the indicated antibodies followed by the appropriate secondary antibodies conjugated to horseradish peroxidase (KPL, Gaithersburg, MD). Immunoreactive bands were visualized using enhanced chemiluminescence (Pierce). The molecular sizes of the proteins detected were determined by comparison with prestained protein markers (Invitrogen).

### Statistical analysis

The statistical analysis was performed with SPSS software (version 17.0 for Windows). Results are presented as mean ± S.D. Analysis of variance and the Tukey-Kramer multiple-comparison test were used in comparisons. *P* < 0.05 was considered statistically significant.

## SUPPLEMENTARY MATERIALS FIGURES AND TABLES



## Abbreviations

2-DG: 2-Deoxyglucose
JNK: c-Jun NH2-terminal Kinase
PA: phosphatidic acid

## References

[1] Shangary S, Johnson D (2003). Recent advances in the development of anticancer agents targeting cell death inhibitors in the Bcl-2 protein family. Leukemia.

[2] Gauthier ER, Piche L, Lemieux G, Lemieux R (1996). Role of bcl-X(L) in the control of apoptosis in murine myeloma cells. Cancer Res.

[3] Nie C, Luo Y, Zhao X, Luo N, Tong A, Liu X, Yuan Z, Wang C, Wei Y (2014). Caspase-9 mediates Puma activation in UCN-01-induced apoptosis. Cell Death Dis.

[4] Vogler M, Butterworth M, Majid A, Walewska RJ, Sun XM, Dyer MJ, Cohen GM (2009). Concurrent up-regulation of BCL-XL and BCL2A1 induces approximately 1000-fold resistance to ABT-737 in chronic lymphocytic leukemia. Blood.

[5] Choudhary G, Al-Harbi S, Mazumder S, Hill B, Smith M, Bodo J, Hsi E, Almasan A (2015). MCL-1 and BCL-xL-dependent resistance to the BCL-2 inhibitor ABT-199 can be overcome by preventing PI3K/AKT/mTOR activation in lymphoid malignancies. Cell Death Dis.

[6] Mérino D, Khaw SL, Glaser SP, Anderson DJ, Belmont LD, Wong C, Yue P, Robati M, Phipson B, Fairlie WD (2012). Bcl-2, Bcl-xL, and Bcl-w are not equivalent targets of ABT-737 and navitoclax (ABT-263) in lymphoid and leukemic cells. Blood.

[7] Wong M, Tan N, Zha J, Peale FV, Yue P, Fairbrother WJ, Belmont LD (2012). Navitoclax (ABT-263) reduces Bcl-xL–mediated chemoresistance in ovarian cancer models. Mol Cancer Ther.

[8] Schoenwaelder SM, Jarman KE, Gardiner EE, Qiao J, White MJ, Josefsson EC, Alwis I, Ono A, Willcox A, Andrews RK (2011). Bcl-xL–inhibitory BH3 mimetics can induce a transient thrombocytopathy that undermines the hemostatic function of platelets. Blood.

[9] Souers AJ, Leverson JD, Boghaert ER, Ackler SL, Catron ND, Chen J, Dayton BD, Ding H, Enschede SH, Fairbrother WJ (2013). ABT-199, a potent and selective BCL-2 inhibitor, achieves antitumor activity while sparing platelets. Nat Med.

[10] Pan R, Hogdal LJ, Benito JM, Bucci D, Han L, Borthakur G, Cortes J, DeAngelo DJ, Debose L, Mu H (2014). Selective BCL-2 inhibition by ABT-199 causes on-target cell death in acute myeloid leukemia. Cancer Discov.

[11] Chonghaile TN, Roderick JE, Glenfield C, Ryan J, Sallan SE, Silverman LB, Loh ML, Hunger SP, Wood B, DeAngelo DJ (2014). Maturation stage of T-cell acute lymphoblastic leukemia determines BCL-2 versus BCL-XL dependence and sensitivity to ABT-199. Cancer Discov.

[12] Touzeau C, Dousset C, Le Gouill S, Sampath D, Leverson J, Souers A, Maiga S, Bene M, Moreau P, Pellat-Deceunynck C (2014). The Bcl-2 specific BH3 mimetic ABT-199: a promising targeted therapy for t (11; 14) multiple myeloma. Leukemia.

[13] Niu X, Wang G, Wang Y, Caldwell JT, Edwards H, Xie C, Taub JW, Li C, Lin H, Ge Y (2014). Acute myeloid leukemia cells harboring MLL fusion genes or with the acute promyelocytic leukemia phenotype are sensitive to the Bcl-2-selective inhibitor ABT-199. Leukemia.

[14] Coloff JL, Macintyre AN, Nichols AG, Liu T, Gallo CA, Plas DR, Rathmell JC (2011). Akt-dependent glucose metabolism promotes Mcl-1 synthesis to maintain cell survival and resistance to Bcl-2 inhibition. Cancer Res.

[15] Meynet O, Beneteau M, Jacquin M, Pradelli L, Cornille A, Carles M, Ricci J (2012). Glycolysis inhibition targets Mcl-1 to restore sensitivity of lymphoma cells to ABT-737-induced apoptosis. Leukemia.

[16] Fang Y, Vilella-Bach M, Bachmann R, Flanigan A, Chen J (2001). Phosphatidic acid-mediated mitogenic activation of mTOR signaling. Science.

[17] Gong R, Park CS, Abbassi NR, Tang SJ (2006). Roles of glutamate receptors and the mammalian target of rapamycin (mTOR) signaling pathway in activity-dependent dendritic protein synthesis in hippocampal neurons. J Biol Chem.

[18] Bjorkoy G, Lamark T, Brech A, Outzen H, Perander M, Overvatn A, Stenmark H, Johansen T (2005). p62/SQSTM1 forms protein aggregates degraded by autophagy and has a protective effect on huntingtin-induced cell death. J Cell Biol.

[19] Shanware NP, Bray K, Eng CH, Wang F, Follettie M, Myers J, Fantin VR, Abraham RT (2014). Glutamine deprivation stimulates mTOR-JNK-dependent chemokine secretion. Nat Commun.

[20] Kato H, Nakajima S, Saito Y, Takahashi S, Katoh R, Kitamura M (2012). mTORC1 serves ER stress-triggered apoptosis via selective activation of the IRE1–JNK pathway. Cell Death Differ.

[21] Xi H, Kurtoglu M, Liu H, Wangpaichitr M, You M, Liu X, Savaraj N, Lampidis TJ (2011). 2-Deoxy-D-glucose activates autophagy via endoplasmic reticulum stress rather than ATP depletion. Cancer Chemother Pharmacol.

[22] Cang S, Iragavarapu C, Savooji J, Song Y, Liu D (2015). ABT-199 (venetoclax) and BCL-2 inhibitors in clinical development. J Hematol Oncol.

[23] Bodo J, Zhao X, Smith MR, Hsi ED (2014). Activity of ABT-199 and acquired resistance in follicular lymphoma cells. Blood.

[24] Grethe S, Ares MP, Andersson T, Pörn-Ares MI (2004). p38 MAPK mediates TNF-induced apoptosis in endothelial cells via phosphorylation and downregulation of Bcl-x L. Exp Cell Res.

[25] Wilkins JM, McConnell C, Tipton PA, Hannink M (2014). A conserved motif mediates both multimer formation and allosteric activation of phosphoglycerate mutase 5. J Biol Chem.

[26] Upreti M, Galitovskaya EN, Chu R, Tackett AJ, Terrano DT, GranellS Chambers TC (2008). Identification of the major phosphorylation site in Bcl-xL induced by microtubule inhibitors and analysis of its functional significance. J Biol Chem.

[27] Zhao Y, Liu H, Liu Z, Ding Y, LeDoux SP, Wilson GL, Voellmy R, Lin Y, Lin W, Nahta R (2011). Overcoming trastuzumab resistance in breast cancer by targeting dysregulated glucose metabolism. Cancer Res.

[28] Suh DH, Kim MK, No JH, Chung HH, Song YS (2011). Metabolic approaches to overcoming chemoresistance in ovarian cancer. Ann N Y Acad Sci.

[29] Lin YG, Shen J, Yoo E, Liu R, Yen HY, Mehta A, Rajaei A, Yang W, Mhawech-Fauceglia P, DeMayo FJ (2015). Targeting the glucose-regulated protein-78 abrogates Pten-null driven AKT activation and endometrioid tumorigenesis. Oncogene.

[30] Zhao Y, Butler EB, Tan M (2013). Targeting cellular metabolism to improve cancer therapeutics. Cell Death Dis.

[31] Stamelos VA, Redman CW, Richardson A (2012). Understanding sensitivity to BH3 mimetics: ABT-737 as a case study to foresee the complexities of personalized medicine. J Mol Signal.

[32] Halliez M, Maïga S, Touzeau C, Gomez-Bougie P, Le Gouill S, Pellat-Deceunynck C, Amiot M (2013). Abstract C48: Dual targeting of myeloma cells by 2-deoxy-D-glucose and ABT-199 combination respectively through the down-regulation of Mcl-1 and binding to Bcl-2. Mol Cancer Ther.

[33] Maïga S, Touzeau C, Gomez-Bougie P, Le Gouill S, P Marie-Christine Bene, Moreau P, Pellat-Deceunynck C, Amiot M (2013). Combination Of 2-Deoxy-D-Glucose With ABT-199 Efficiently Kills All Molecular Subgroups Of Myeloma Cells. Blood.

[34] Pradelli L, Beneteau M, Chauvin C, Jacquin M, Marchetti S, Munoz-Pinedo C, Auberger P, Pende M, Ricci J (2010). Glycolysis inhibition sensitizes tumor cells to death receptors-induced apoptosis by AMP kinase activation leading to Mcl-1 block in translation. Oncogene.

[35] Yamaguchi R, Janssen E, Perkins G, Ellisman M, Kitada S, Reed JC (2011). Efficient elimination of cancer cells by deoxyglucose-ABT-263/737 combination therapy. Plos One.

[36] Ramírez-Peinado S, Alcázar-Limones F, Lagares-Tena L, El Mjiyad N, Caro-Maldonado A, Tirado OM, Muñoz-Pinedo C (2011). 2-deoxyglucose induces Noxa-dependent apoptosis in alveolar rhabdomyosarcoma. Cancer Res.

[37] Hikita H, Takehara T, Shimizu S, Kodama T, Shigekawa M, Iwase K, Hosui A, Miyagi T, Tatsumi T, Ishida H, Li W, Kanto T, Hiramatsu N (2010). The Bcl-xL inhibitor, ABT-737, efficiently induces apoptosis and suppresses growth of hepatoma cells in combination with sorafenib. Hepatology.

[38] Kelkel M, Cerella C, Mack F, Schneider T, Jacob C, Schumacher M, Dicato M, Diederich M (2012). ROS-independent JNK activation and multisite phosphorylation of Bcl-2 link diallyl tetrasulfide-induced mitotic arrest to apoptosis. Carcinogenesis.

[39] Fan M, Goodwin M, Vu T, Brantley-Finley C, Gaarde WA, Chambers TC (2000). Vinblastine-induced phosphorylation of Bcl-2 and Bcl-XL is mediated by JNK and occurs in parallel with inactivation of the Raf-1/MEK/ERK cascade. J Biol Chem.

[40] Garami A, Zwartkruis FJ, Nobukuni T, Joaquin M, Roccio M, Stocker H, Kozma SC, Hafen E, Bos JL, Thomas G (2003). Insulin activation of Rheb, a mediator of mTOR/S6K/4E-BP signaling, is inhibited by TSC1 and 2. Mol Cell.

[41] Maiuri MC, Le Toumelin G, Criollo A, Rain JC, Gautier F, Juin P, Tasdemir E, Pierron G, Troulinaki K, Tavernarakis N (2007). Functional and physical interaction between Bcl-XL and a BH3-like domain in Beclin-1. EMBO J.

[42] Jin Z, Li Y, Pitti R, Lawrence D, Pham VC, Lill JR, Ashkenazi A (2009). Cullin3-based polyubiquitination and p62-dependent aggregation of caspase-8 mediate extrinsic apoptosis signaling. Cell.

[43] Huang DC, Cory S, Strasser A (1997). Bcl-2, Bcl-XL and adenovirus protein E1B19kD are functionally equivalent in their ability to inhibit cell death. Oncogene.

[44] Jeong SY, Gaume B, Lee YJ, Hsu YT, Ryu SW, Yoon SH, Youle RJ (2004). Bcl-x(L) sequesters its C-terminal membrane anchor in soluble, cytosolic homodimers. EMBO J.

[45] Shen W, Du R, Li J, Luo X, Zhao S, Chang A, Zhou W, Gao R, Luo D, Wang J (2016). TIFA suppresses hepatocellular carcinoma progression via MALT1-dependent and-independent signaling pathways. Signal Transduction and Targeted Therapy.

[46] Guo W, Zhang Y, Ling Z, Liu X, Zhao X, Yuan Z, Nie C, Wei Y (2015). Caspase-3 feedback loop enhances Bid-induced AIF/endoG and Bak activation in Bax and p53-independent manner. Cell Death Dis.

[47] Hu W, Wang F, Tang J, Liu X, Yuan Z, Nie C, Wei Y (2012). Proapoptotic protein Smac mediates apoptosis in cisplatin-resistant ovarian cancer cells when treated with the anti-tumor agent AT101. J Biol Chem.

