# Supplementary material for: Mcl-1 expression and JNK activation induces a threshold for apoptosis in Bcl-xL-overexpressing hematopoietic cells
Source: Oncotarget. 2016 Dec 26;8(7):11042–52. doi: 10.18632/oncotarget.14223 (PMC5355244; doi:10.18632/oncotarget.14223)
Supplement: Supplementary file 1 [file oncotarget-08-11042-s001.pdf]

Mcl-1 expression and JNK activation induces a threshold for apoptosis in Bcl-xL-overexpressing hematopoietic cells

Supplementary Materials

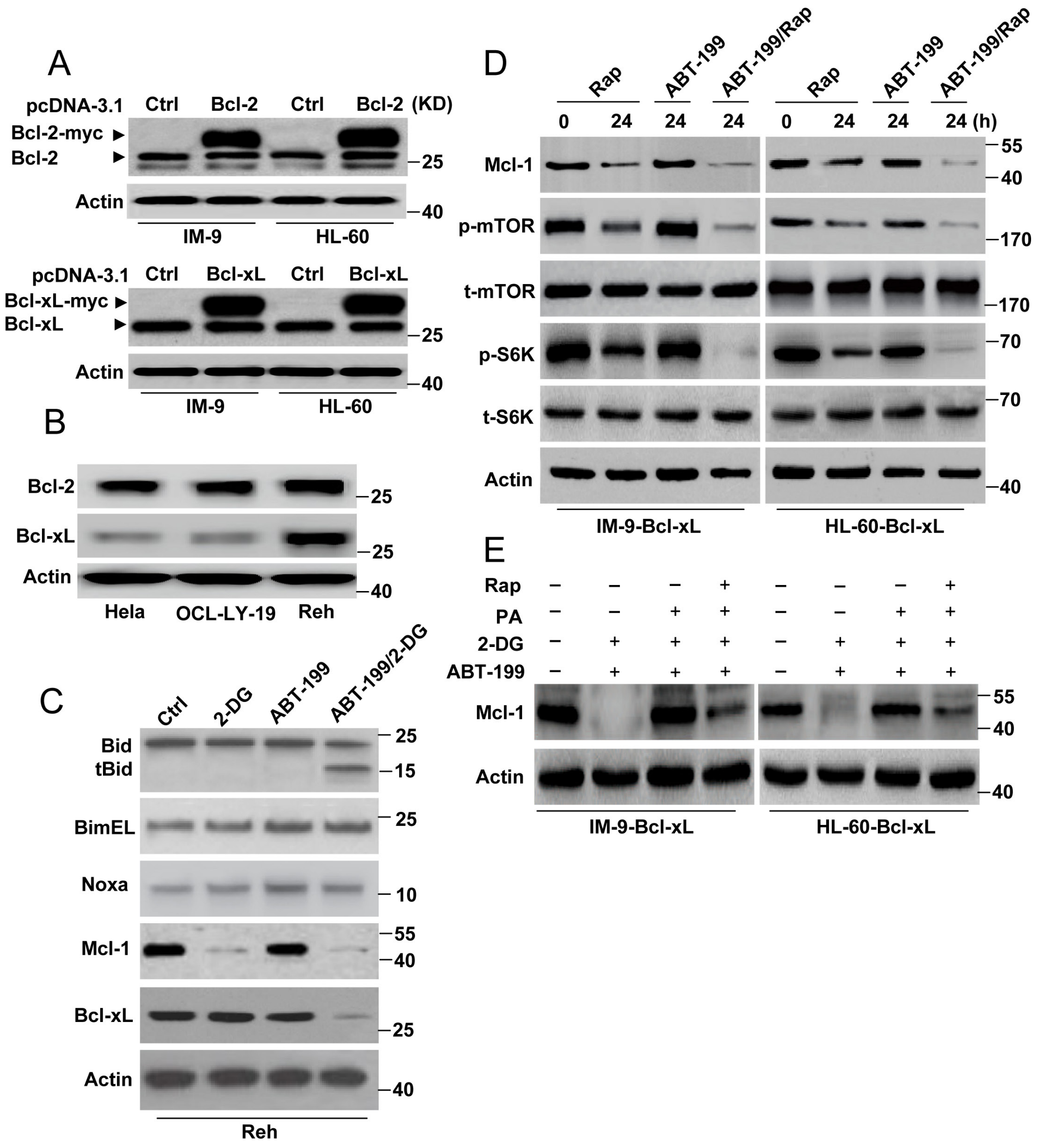

Supplementary Figure S1: (**A**) Cells were stably transfected with Ctrl, pcDNA3.1-Bcl-2, p cDNA3.1-Bcl-xL vector and collected for western blot detection. β-Actin was used as a protein loading control. (**B**) Indicated cells were collected for western blot detection. (**C**) Reh cells were treated with ABT-199 for 24 h, and then treated cells were collected for apoptosis detection. Graphs showing results of quantitative analyses (n = 3, mean ± S.D. $^{**}P < 0.01$). (**D**) IM-9-Bcl-xL and HL-60-Bcl-xL were treated with ABT-199 (50 nM), rapamycin (25 nM) or the indicated combination treatments for 24 h. Treated cells were lysed for immunoblot detection. (**E**) Cells were subjected to the indicated combination treatment (2-DG, 5 mM; ABT-199, 50 nM; PA, 100 μM; rapamycin, 25 nM) for 24 h, and then lysed for immunoblot detection. Representative results of three experiments with consistent results are shown.

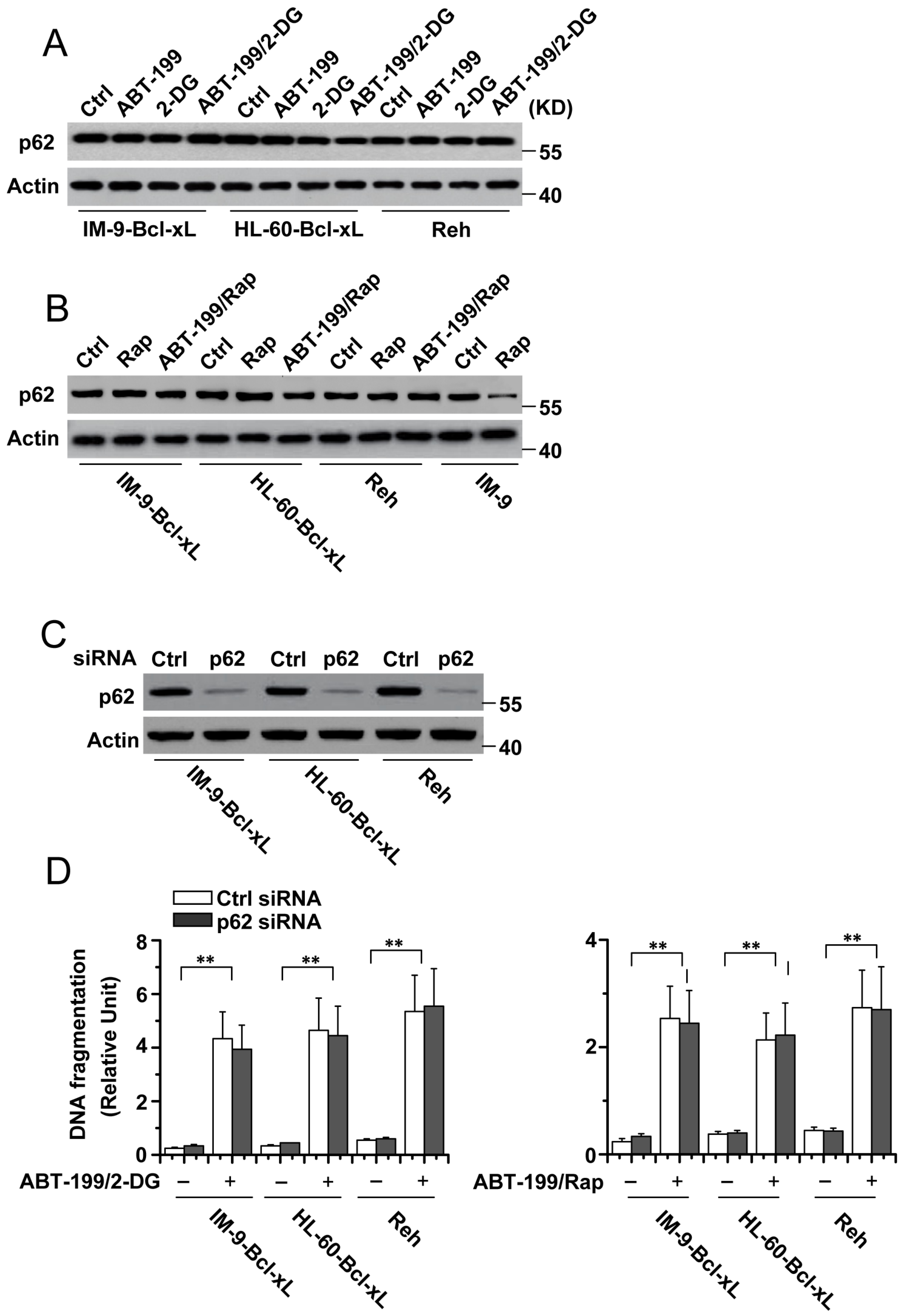

Supplementary Figure S2: (**A**) Indicated cells were treated with 2-DG (5 mM), ABT-199 (50 nM) or the indicated combination treatment for 24 h. Treated cells were lysed for detecting p62 expression. β-Actin was used as a protein loading control. (**B**) Indicated cells were treated with ABT-199 (50 nM), rapamycin (25 nM) or the indicated combination treatment for 24 h. Treated cells were lysed for detecting p62 expression. (**C**) Cells were transfected with Ctrl or p62 siRNA for 48 h, and then cells were collected for p62 expression detection. (**D**) Cells were transfected with Ctrl or p62 siRNA as C, and then treated with ABT-199/2-DG or ABT-199/Rap for 24 h. Treated cells were collected for apoptosis detection. Graphs showing results of quantitative analyses (n = 3, mean ± S.D. **P < 0.01).

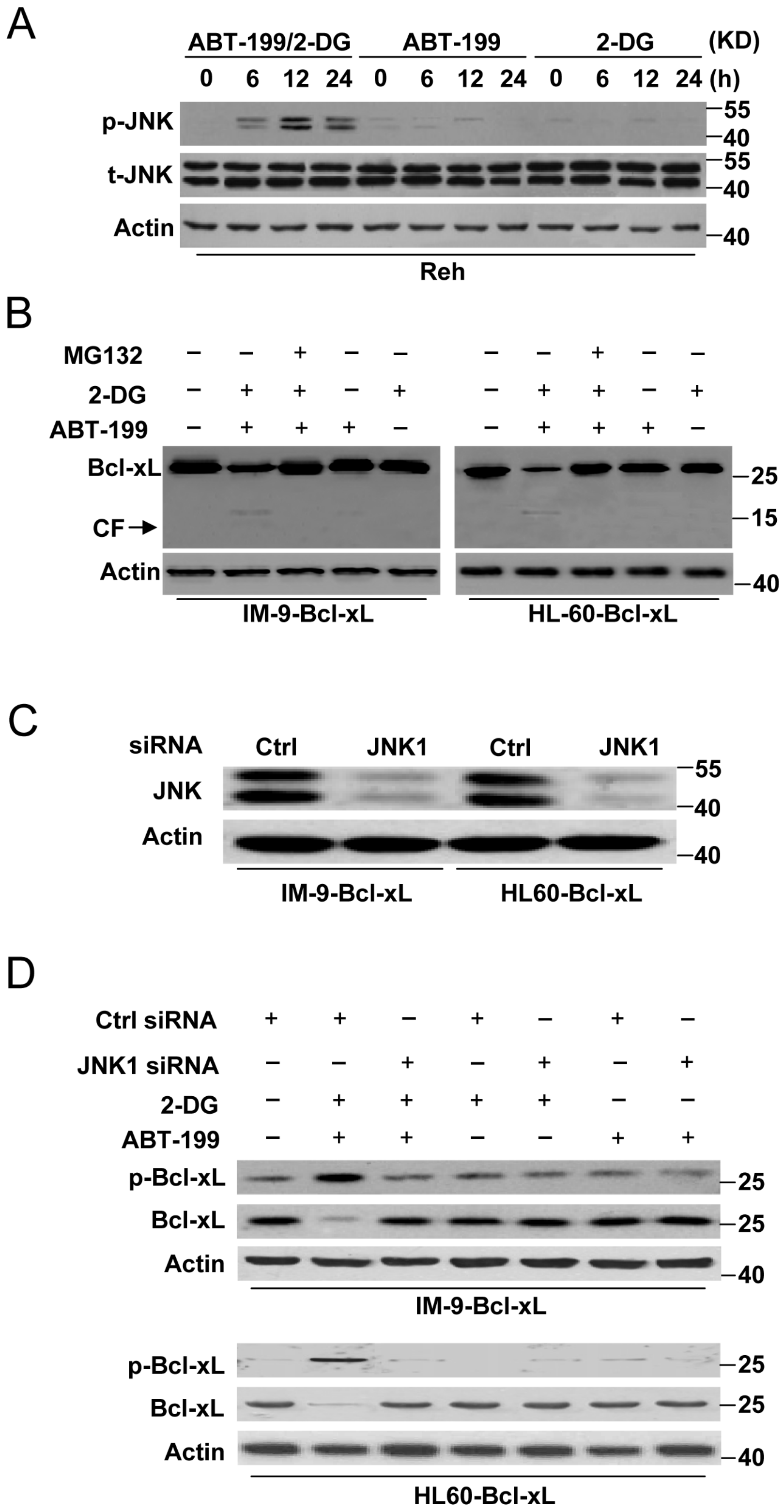

Supplementary Figure S3: (**A**) Cells were treated with 2-DG (5 mM), ABT-199 (50 nM) or the combination of the two (2-DG, 5 mM; ABT-199, 50 nM) at the indicated time points, and then lysed for western blot detection. β-Actin was used as a protein loading control. (**B**) Cells were treated with 2-DG (5 mM), ABT-199 (50 nM), MG-132 (1 μM) or the indicated combination treatment for 24 h, and then lysed for western blot detection. (**C**) Cells were transfected with JNK1 siRNA or Ctrl siRNA for 48 h, and then lysed for western blot detection. (**D**) Cells were transfected with JNK1 siRNA or Ctrl siRNA for 48 h, and then treated as the indicated and then analyzed for immunoblot. All data are representative of three independent experiments.

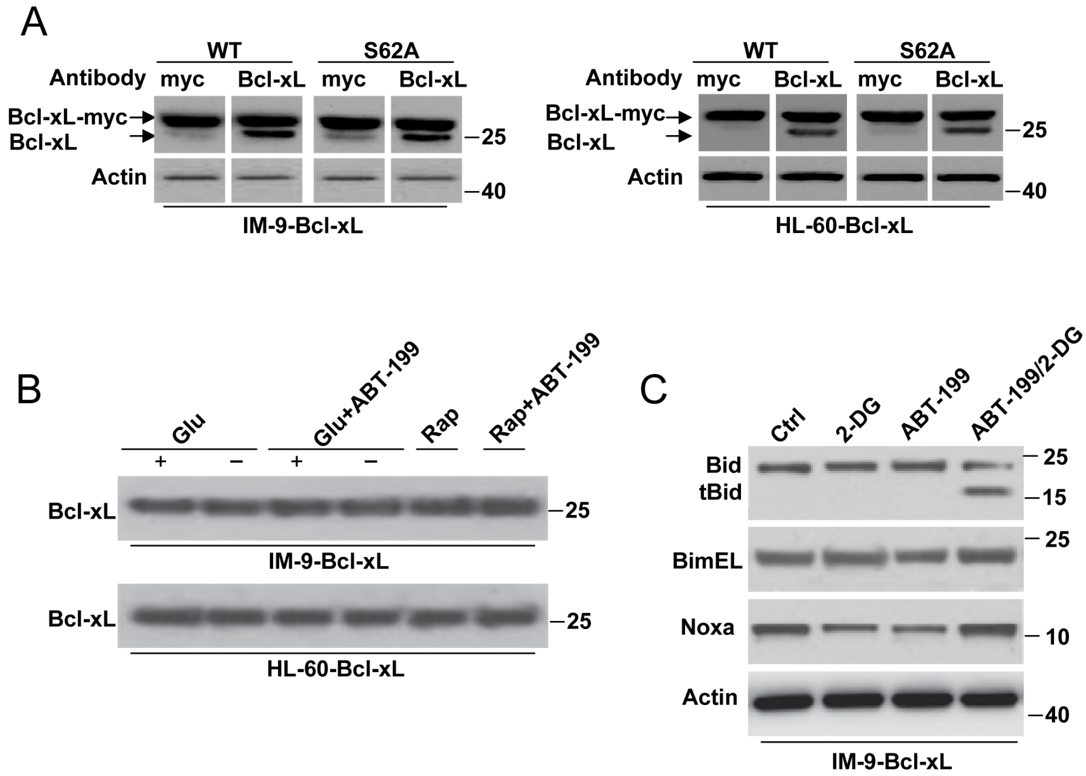

Supplementary Figure S4: (**A**) Cells were transfected with Bcl-xL-Myc or Bcl-xL-S62A-Myc for 48 h, and then lysed for western blot detection. β-Actin was used as a protein loading control. (**B**) Cells were treated as the indicated and then analyzed for immunoblot. (**C**) IM-9/Bcl-xL cells were treated with 2-DG, ABT-199 or the indicated combination treatment for 24 h, and then lysed for western blot detection. Representative results of three experiments with consistent results are shown.

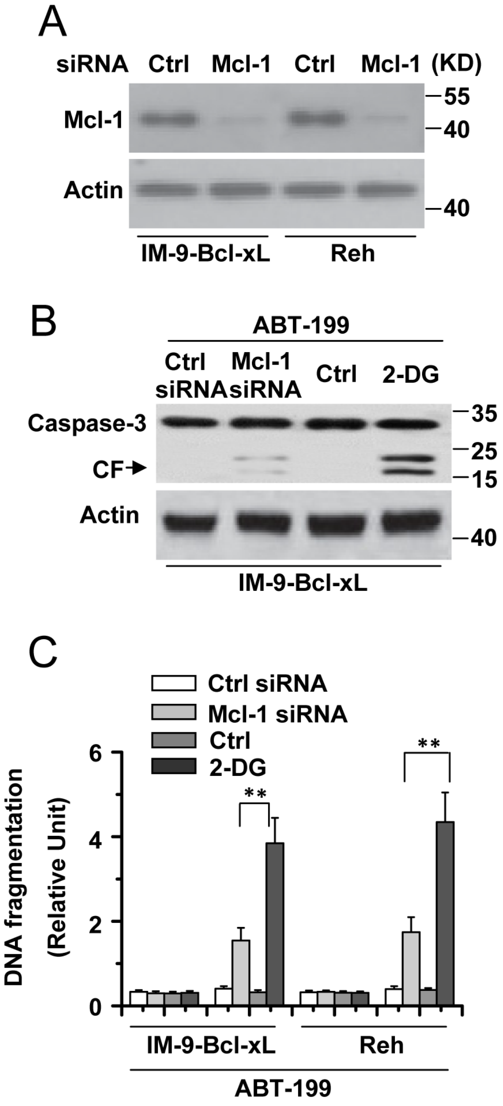

Supplementary Figure S5: (**A**) Indicated cells were transfected with Mcl-1 or Ctrl siRNA for 48 h, and then lysed for western blot detection. β-Actin was used as a protein loading control. (**B**) One portion of IM-9/Bcl-xL cells was transfected with Mcl-1 or Ctrl siRNA for 48 h, and then treated with ABT-199 for 24 h. the other portion of cells were treated with ABT-199 and 2-DG for 24 h. All treated cells were lysed for western blot detection. (**C**) Cells were treated as described in B, and then treated cells were collected for apoptosis detection. Graphs showing results of quantitative analyses ($n = 3$, mean ± S.D. $**P < 0.01$).

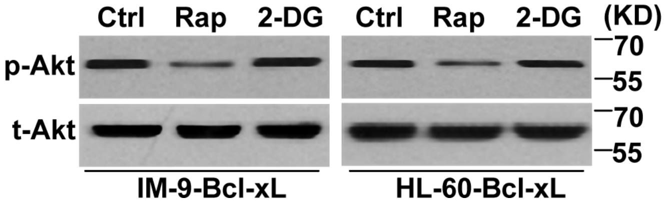

Supplementary Figure S6: Cells were treated with 2-DG (5 mM), or rapamycin (25 nM) for 24 h, and then analyzed for immunoblot. All data are representative of three independent experiments.
